# Comparison of Low-Versus High-Dose Steroids in the Clinical Outcome of Hospitalized COVID-19 Patients

**DOI:** 10.3390/antibiotics10121510

**Published:** 2021-12-09

**Authors:** Zubia Jamil, Fahad N. Almajhdi, Samreen Khalid, Muhammad Asghar, Jamal Ahmed, Yasir Waheed

**Affiliations:** 1 Department of Medicine, Foundation University Medical College, Foundation University Islamabad, Islamabad 44000, Pakistan; zubiajamil321@gmail.com (Z.J.); samreendoctor@gmail.com (S.K.); 2 Department of Botany and Microbiology, College of Science, King Saud University, Riyadh 11451, Saudi Arabia; majhdi@ksu.edu.sa; 3 Division of Infectious Diseases, Department of Medicine Solna, Karolinska Instututet, 17177 Stockholm, Sweden; asghar.muhammad@ki.se; 4 Department of Infectious Diseases, Karolinska University Hospital, 17164 Stockholm, Sweden; 5 Department of Pulmonology, Fauji Foundation Hospital, Rawalpindi 45000, Pakistan; Jamal593@gmail.com; 6 Multidisciplinary Lab, Foundation University Medical College, Foundation University Islamabad, Islamabad 44000, Pakistan

**Keywords:** COVID-19, Dexamethasone, Methylprednisolone, cytokine release syndrome, steroids

## Abstract

(1) Objectives: Patients with COVID-19 infection have been given various formulations and dosages of steroids over the last year and a half. This study aims to compare the effects of different formulations and doses of steroids on the 30 day in-hospital clinical outcome of patients with severe COVID-19 infection. (2) Material and Methods: An analysis of a retrospective cohort was carried out on patients with severe COVID-19 infection in a high-dependency unit (HDU) between February and July 2021. In total, 557 patients were included in this study. Patients who did not receive steroids (124) were excluded. Patients were divided into three groups based on dosages of steroids (Dexamethasone = 6 mg/day, Dexamethasone > 6 mg/day, and Methylprednisolone = 500 mg/day), given for 10 days. First, clinical outcome was evaluated on the 10th day of steroid administration in relation to mode of oxygen delivery. Then, Kaplan–Meier analysis was employed to determine 30 day in-hospital survival in relation to the use of steroid. (3) Results: Three groups were statistically equal according to biochemical characteristics. After 10 days of Methylprednisolone = 500 mg/day vs. Dexamethasone = 6 mg/day, 10.9% vs. 6.2% of patients required invasive ventilation (*p* = 0.01). The 30 day in-hospital mortality was lowest, 3%, in individuals receiving Dexamethasone = 6 mg/day, compared to 3.9% in individuals receiving Dexamethasone > 6 mg/day and 9.9% in individuals receiving Methylprednisolone = 500 mg/day, respectively. The median elapsed time was longer than 28 days between admission and outcome for Dexamethasone = 6 mg/day, compared to 18 days for Dexamethasone > 6 mg/day and 17 days for Methylprednisolone = 500 mg/day (*p* = < 0.0001). Dexamethasone = 6 mg/day was found to be a positive predictor of clinical outcome in COVID-19 patients on regression analysis. (4) Conclusions: Low-dose Dexamethasone (6 mg/day) is more effective than high-dose Dexamethasone and Methylprednisolone in improving the survival outcome of severe COVID-19 cases.

## 1. Introduction

COVID-19 infection (SARS-CoV2) is a worldwide pandemic with a high mortality rate that poses a huge threat to the human population and has led to a severe medical crisis, thereby rendering diagnostic and treatment strategies a crucial requirement to combat the crisis [1,2,3]. Infection with the virus can range in severity from mild to severe and even result in death [4]. Older individuals with comorbidities are more prone to contract the disease, with worse prognosis and fatal outcomes [5].

Cytokine storm syndrome and systemic inflammatory response triggered by SARS-CoV-2 play a crucial role in conferring pathogenicity during infection [6]. Patients with severe COVID-19 infection develop hyperactivation of the immune system, resulting in cytokine release, leading to endothelial lung injury and microvascular thrombosis. This not only precipitates severe lung damage but also decreases organ perfusion, resulting in multi-organ failure [7]. Studies have suggested that corticosteroids have potent anti-inflammatory effects that help reduce hyperimmune response in severe COVID-19 infection [8].

The highly contagious potential of this virus is posing a tremendous threat worldwide, affecting every aspect of life. Scientists around the world are working relentlessly at full capacity to explore effective treatment strategies to deal with the calamity. Reducing transmission by preventive measures is the most practical and efficient method to control the disease process [9]. 

In the amidst of this crisis, it has become an immediate need to develop effective management against COVID-19 with its deleterious effects in causing cytokine storm syndrome and systemic inflammatory response [10]. Evidence shows that low-dose systemic glucocorticoids and Heparin may control inflammation-mediated lung injury and mitigate the effects of cytokine storm syndrome, thereby preventing progression to respiratory failure and death [8,11].

Using data from large multi-center, randomized controlled trials, the WHO and The National Institute for Health strongly recommend that severe COVID-19 infection needing sustained respiratory support be treated with low-dose systemic steroids for up to 10 days [12]. Administration of low-dose Dexamethasone has been proved to reduce the duration of invasive mechanical ventilation and in-hospital mortality in severe and critically ill COVID-19 patients [13].

Over the last year and a half of the COVID-19 pandemic, various formulations and dosages of steroids have been used on patients with COVID-19 infection. The purpose of our study is to compare differences in the 30 day in-hospital clinical outcome of patients with severe COVID-19 pneumonia treated with low-dose Dexamethasone, high-dose Dexamethasone and Methylprednisolone. The necessity of invasive mechanical ventilation and changes in oxygen requirements are considered secondary outcomes.

## 2. Materials and Methods

### 2.1. Study Design and Settings

In the last year and a half of the COVID-19 pandemic, different formulations and dosages of steroids were extensively used in severe COVID-19 patients admitted to the HDU of Foundation University Hospital Islamabad, Pakistan. So, we conducted a retrospective cohort analysis on individuals with severe COVID-19 infection who required admission to the HDU of Foundation University Islamabad’s teaching hospital from the first week of February 2021 to July 2021 to estimate the efficacy of diverse formulations and dosages of steroids. In response to the COVID-19 pandemic, our hospital designated the 32-bed high-dependency unit, fully equipped with all the requirements including invasive mechanical ventilation, to deal with patients presenting with hypoxemia.

The key aim of this research was assessment of the efficacy of different formulations along with different dosages of steroids on the 30 day in-hospital outcome of severe COVID-19 patients admitted to the HDU.

### 2.2. Participants Characteristics

Study participants included the following patients:

1. Patients who had COVID-19 infection confirmed by reverse transcription polymerase chain reaction (RT-PCR) and required admission to the high-dependency unit (HDU).
2. CRITICAL disease: Evidence of ARDS on ABGs (mild: PaO_2_/FiO_2_ > 200 but < 300 mmHg; moderate: PaO_2_/FiO_2_ > 100 but < 200 mmHg; severe: PaO_2_/FiO_2_ < 100 mmHg) + multi-organ involvement + septic shock.
3. SEVERE disease: Infiltrates > 50% of total lung fields on chest X ray or HRCT chest showing extensive peripheral ground glass opacities + SpO2 < 90% + RR > 30/min + BP < 90 mmHg with tachycardia.

The following patients were excluded.

1. Patients who did not receive steroids.
2. Patients who had contraindication to use of steroids (systemic fungal infection, active concomitant tuberculosis, documented hypersensitivity reaction to intravenous steroids, uncontrolled blood pressure, and uncontrolled diabetes status).
3. Severe congestive cardiac failure (EF < 25%).
4. Death within 48 h of admission.
5. Patients who were receiving other investigational therapies simultaneously such as tocilizumab and plasmapheresis.

### 2.3. Formulation and Dosages of Steroids

Three formulations and dosages of steroids (Dexamethasone 6 mg/day, Dexamethasone > 6 mg/day, and Methylprednisolone 500 mg/day) were given for 10 days to COVID-19 patients admitted to the HDU. So, the study cohort was divided as follows:

1. Patients who received Dexamethasone 6 mg/day.
2. Patients who were given Dexamethasone > 6 mg/day.
3. Patients who received Methylprednisolone 500 mg/day.

### 2.4. Methodology

Data on patients in this tertiary care hospital are maintained and saved in MEDIX medical software system. Every has a specific MR number, used to trace history, hospital management and laboratory test results. Before retrieving patient data, ethical approval was granted from the Ethical Committee of our hospital (Letter No. 506/RC/FFH/RWP 20 January 2021). The individual identities of patients were fully concealed by the hospital before acquisition. At the time of admission to the HDU, informed consent was signed by every patient or their attendant detailing that their patient data can be utilized in studies on COVID-19 infection by maintaining full privacy and integrity of each patient. 

All patients with severe COVID-19 admitted from first week of February 2021 to July 2021 were tracked. First, laboratory parameters were noted and analyzed for each group followed by clinical outcome on the 10th day of administration of steroids in relation to mode of oxygen delivery and at 30 day of HDU admission in relation to the end point of this study (survived or died). Patients who were not given steroids were excluded as the primary aim of this study was to compare the effectiveness of different formulations along with different dosages of steroids on the outcome of severe COVID-19 patients. 

### 2.5. Statistical Analysis

Specifically, for quantitative variables, the mean, standard deviation, and range were used, while for qualitative variables, the percentage was used. One-way ANOVA was employed to compare quantitative variables among three groups; chi-square tests were utilized to compare qualitative variables (SPSS version 26) (IBM Corp. IBM SPSS Statistics for Windows, Version 26.0, Armonk, New York, NY, USA). First, clinical outcome was compared at the time of admission to 10th day of steroid administration in relation to mode of oxygen delivery by contingency co-efficient. To analyze the 30 day in-hospital outcome in relation to the use of different formulations and dosages of steroids, log-rank and Kaplan–Meier analyses were used by MedCalc Statistical Software 19.6.4 (MedCalc Software, Ostend, Belgium). Cox regression analysis was carried out to evaluate the independent predictors.

## 3. Results

### 3.1. Study Cohort Characteristics

Five hundred and fifty-seven (557) patients with severe COVID-19 infection were admission to the high-dependency unit (HDU) at the Fauji Foundation Hospital, Rawalpindi during the study period. The purpose of this study was to compare the efficacy of different steroid dosages and various formulations.

This study included 557 COVID-19 patients, of which four hundred and thirty-three (*n* = 433) patients were given steroids and one hundred and twenty-four (*n* = 124) did not receive steroids due to contraindications, so they were excluded from this study. 

The mean age of 433 patients receiving steroids was 55.94 + 16.99 (14–88) years; females 76.9% (*n* = 333) vs. males 23.1% (*n* = 100).

Results showed that majority of COVID-19 patients who required oxygen inhalation and HDU admission had multiple co-morbidities 78.3% (*n* = 339). Diabetes mellitus was the most common co-morbid condition 60% (*n* = 260) in this study cohort. Other co-morbidities were hypertension 55.7% (*n* = 241), chronic kidney disease 12.9% (*n* = 56), cerebrovascular accidents 10.4% (*n* = 45), ischemic heart disease 9.7% (*n* = 42), chronic obstructive pulmonary disease 8.8% (*n* = 38), congestive cardiac failure 4.6% (*n* = 20), use of immunosuppressive drugs 4.6% (*n* = 20), presence of solid tumors 4.2% (*n* = 18) and chronic liver disease 3.9% (*n* = 17). 

Patients who received steroids (*n* = 433) were divided into three groups according to the formulation and dosage of steroids used.

1. Patients receiving Dexamethasone = 6 mg/day, 40.6% (*n* = 176).
2. Patients receiving Dexamethasone > 6 mg/day, 11.1% (*n* = 48).
3. Patients receiving Methylprednisolone = 500 mg/day, 48.3% (*n* = 209).

When we compared the biochemical parameters of the three groups of COVID-19 patients, we found that the three groups were not statistically different from each other. The biochemical characteristics of the three groups of COVID-19 patients at the time of admission to the HDU in relation to use of steroids are given in Table 1.

### 3.2. Outcome in Related to Mode of Oxygen Delivery

Among 433 patients, 83.1% (360/433) survived and 16.9% (73/433) did not survive during HDU admission. When comparing the mode of oxygen delivery at the time of admission and after 10 days of administration of different dosages and formulation of steroids, we found that Dexamethasone 6 mg/day group required only 6.2% invasive ventilation after 10 days compared to the Methylprednisolone 500 mg/day group, which required 10.9% invasive ventilation (RR = 6.11, 95% CI = 0.96–8.51, *p* = 0.01). Table 2 shows the comparison of different modes of oxygen delivery at the time of admission to the HDU to 10 days treatment of the three groups of steroids (Dexamethasone 6 mg/day, Dexamethasone > 6 mg/day, and Methylprednisolone 500 mg/day).

### 3.3. Survival Analysis

**Figure 1:**
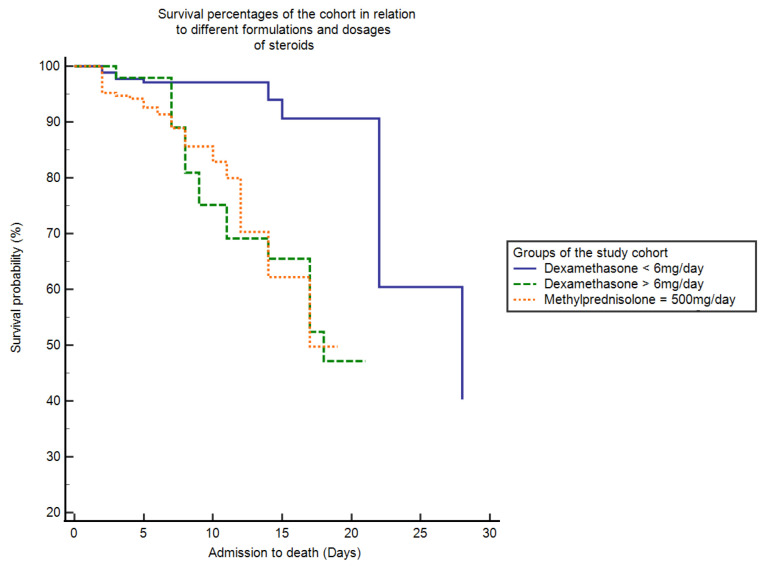
Kaplan–Meier curve which represents the general survival probability from admission to outcome (death) when using different steroid formulations and doses. Log-rank analysis showed that the median time from hospital admission to death was 28 days for the group receiving Dexamethasone 6 mg/day, 18 days for the group receiving Dexamethasone > 6 mg/day and only 17 days for the group receiving Methylprednisolone 500 mg/day (*p* ≤ 0.0001).

The mean duration of HDU stay was 14.08 + 6.15 (8–30) days. The 30 day in hospital mortality was approximately 3% (13/433) in patients receiving Dexamethasone = 6 mg/day, 3.9% (17/433) receiving Dexamethasone > 6 mg/day and 9.9% (43/433) (*p* < 0.0001) in patients receiving Methylprednisolone = 500 mg/day. The median time from hospital admission to death was 28 days for the group receiving Dexamethasone = 6 mg/day (SE = 1.47, 95% CI = 22–28), 18 days for the group receiving Dexamethasone > 6 mg/day (SE = 1.84, 95% CI = 14–18) and 17 days for the group receiving Methylprednisolone 500 mg/day (SE = 1.34, 95% CI = 14–17). The median time from hospital admission to death was significant among three groups (Log rank × 2 = 31.44, *p* ≤ 0.0001). Figure 1 shows the Kaplan–Meier curve which represents the general survival chance from admission to final outcome when using different steroid formulations and doses.

### 3.4. Receiver Operating Characteristic (ROC) Curve

**Figure 2:**
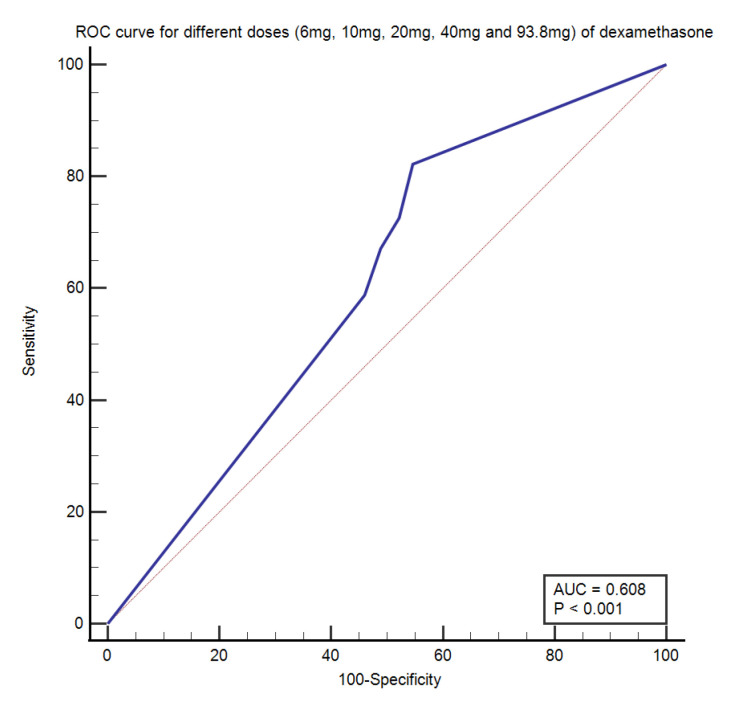
Receiver operating characteristic (ROC) curve showing Dexamethasone = 6 mg, Dexamethasone > 6 mg (10, 20, and 40 mg) and Dexamethasone 93.8 mg (equivalent of Methylprednisolone = 500 mg) in determining the outcome of COVID-19 patients. Cut-off value of 6 mg of Dexamethasone was found by Youden index (*p* ≤ 0.0001).

Dexamethasone = 6 mg, Dexamethasone > 6 mg (10, 20, and 40 mg) and Dexamethasone 93.8 mg (equivalent of Methylprednisolone = 500 mg) was plotted against survival of patients to find the cut-off value among different dosages of steroids through the ROC curve shown in Figure 2. The cut-off value of 6 mg of Dexamethasone was found by Youden index with a sensitivity of 82.19 (95% CI: 71.5–90.2) and a specificity of 45.28 (95% CI: 40.1–50.6) (*p* < 0.001) in determining the survival outcome of COVID-19 patients.

### 3.5. Independent Predictors of Survival

To evaluate the predictors of survival outcome in patients with severe symptoms during stay in the HDU, regression analysis was applied. An initial linear regression analysis was performed to identify statistically significant variables. These predictors were then investigated by Cox-proportional hazard regression analysis. Increasing age, co-morbid conditions (none to presence of ≥1 conditions), a greater number of days requiring oxygen and a greater number of days on ventilator support were found to be influencing the negative outcome in these patients. Among different formulations of steroids, Dexamethasone 6 mg/day was found to be a predictor of clinical outcome in COVID-19 patients. Table 3 shows the Cox-proportional hazard regression analysis for various predictors of outcome (determined by linear regression).

## 4. Discussion

Glucocorticoids exhibit immunosuppressant effects by inhibiting the phagocytic functions of macrophages and reducing the activity as well as number of T lymphocytes but minimally influencing humoral immunity. Methylprednisolone is an intermediate-acting product and it is 5-fold more potent than short-acting products (hydrocortisone), whereas Dexamethasone is a long-acting product and it is 25-fold more potent than short-acting products [14].

The outcomes of this research disclosed that compared to higher doses of Dexamethasone > 6 mg/day or Methylprednisolone 500 mg/d, patients with COVID-19 pneumonia admitted to the HDU had lower 30 day in-hospital mortality when treated with Dexamethasone 6 mg/day for 10 days. The benefit was clearly seen in the respiratory support required after 10 days of treatment with steroids, with few patients requiring invasive mechanical ventilation (6.2%) who had received low-dose Dexamethasone 6 mg/day as compared to high-dose Dexamethasone (7.6%) and Methylprednisolone (10.9%). The large multi-center randomized controlled RECOVERY trial by Horby P et al. [13] clearly shows the benefits of low-dose Dexamethasone in reducing mortality among patients with moderate to severe COVID-19 infection requiring oxygen therapy. Our findings are in line with a previous study by Horby P et al. [13]; however, we retrospectively observed an increase in oxygen requirements and a new need for invasive mechanical ventilation, which was less in patients receiving Dexamethasone 6 mg/day, proving the beneficial role of low-dose steroids. Another study by Wagner. C et al. [15] identified the favorable effects of steroids in reducing mortality and need for invasive ventilation in hospitalized COVID-19 patients.

We compared the different doses and formulations of steroids among 433 patients. A total of 40.6% (*n* = 176) received low-dose Dexamethasone = 6 mg/day, 11.1% (*n* = 48) received high-dose Dexamethasone > 6 mg/day and 48.3% (*n* = 209) received Methylprednisolone = 500 mg/day for 10 days. The median length of stay and respiratory support required after 10 days of treatment with different steroids were assessed. We observed that the length of stay from hospital admission to death was prolonged (28 days), with few patients requiring invasive mechanical ventilation, in those who received low-dose Dexamethasone in comparison with high-dose Dexamethasone (18 days) and Methylprednisolone (17 days). A few studies proposed Dexamethasone to be used in severe COVID-19 patients compared to other steroids, focusing on its role in reducing mortality [16,17,18]. Another ongoing RE-MED trial is testing the superiority of high-dose Dexamethasone over low-dose Dexamethasone in COVID-19 patients with more severe symptoms [19].

The overall case fatality rate in this study was 16.9%, while it was higher, 25%, in a study by Hyun J. et al. [20] and a study by Docherty A et al. quotes 32% ICU mortality [21]. Among the subgroups, the mortality rate was higher in patients receiving Methylprednisolone 9.9% than those who received Dexamethasone in either dose 3% (low dose) and 3.9% (high dose). This finding is supported by the METCOVID trial by Jeronimo C et al. [22] which proved no role of Methylprednisolone in reducing mortality in severe COVID-19 infection. The mortality rate was also high, approximately 30%, in a study by Steinberg K et al. [23] among COVID-19 patients with ARDS receiving Methylprednisolone. High-dose steroids were hazardous for patients with comorbidities and older age in another study and could delay viral clearance [24,25]. On the contrary, emerging evidence supports the role of Methylprednisolone in severe and critical COVID-19 patients [26]. Studies by Pinzon M et al. [27] and Ranjbar K, [28] found high-dose Methylprednisolone to be better than Dexamethasone in improving clinical status and need for invasive ventilation, displaying a shorter recovery time and a decrease in severity of inflammatory markers, in COVID-19 patients. This beneficial effect could be due to the higher lung penetration of Methylprednisolone, thus improving lung compliance more effectively. However, the mortality benefit was not statistically significant in the above-mentioned studies. Similarly, other studies showed an improved clinical outcome with early administration of Methylprednisolone in hypoxic patients with more severe disease [29].

The coronavirus (SARS-CoV-2) pandemic has led to increased mortality worldwide. Successful treatment of COVID-19 infection is a major goal and clinical trials are still on their road to make further improvement in halting this deadly disease. Among immune modulators, steroids play a pivotal role in severe COVID-19 pneumonia with cytokine storm syndrome (CSS) and have been shown to reduce progression to respiratory failure and death [8]. The largest meta-analysis of clinical trials by the World Health Organization (WHO), Rapid Evidence Appraisal for COVID-19 Therapies (REACT) [12], has proved that systemic steroids are effective in reducing in-hospital mortality among severe COVID-19 patients. The WHO has published guidelines for the use of low-dose steroids in severe and critically ill COVID-19 patients, highlighting their benefits in improving survival probability and the need for mechanical ventilation, and against their use in mild to moderate COVID-19 infection [30]. Steroid use in non-severe COVID-19 infection leads to worse clinical outcomes, as shown by a case study [31]. Similarly, in a meta-analysis by Van Paassen J et al. [32], steroids proved to be beneficial in severe COVID-19 infection, reducing mortality and need for mechanical ventilation. Early administration of steroids can decrease the number of days on invasive ventilation in patients with ARDS and reduce mortality as well as increase organ support-free days, proved by other studies [33,34,35]. These facts again show the plausible effects of systemic steroids in enhancing survival probability.

Our study showed that patients who had multiple comorbidities developed more severe disease, and therefore required HDU admission. This is evident from data collected by Zhou F et al. [3] and Brook R et al. [36], showing that due to comorbidities, older-age patients had high inflammatory markers and suffered from more severe COVID-19 infection, thereby having a guarded prognosis. Furthermore, our results showed that advanced age, presence of co-morbid conditions, increased number of days on oxygen support and more days on mechanical ventilator were poor predictors of survival while low.

Dexamethasone 6 mg/day for 10 days improved survival outcomes in patients with severe COVID-19 infection. On the contrary, in the CoDEX trial by Tomazini BM et al. [37], use of 10–20 mg of Dexamethasone along with standard care vs. standard care alone was associated with increased number of ventilator-free days in severe COVID-19 patients with ARDS. However, this trial is different as it is comparing high-dose Dexamethasone with standard care; but in our study, we have compared different doses of steroids.

## 5. Conclusions

To conclude, this study provides evidence that low-dose corticosteroids are beneficial in reducing 30 day in hospital mortality in severe and critically ill COVID-19 patients, with few patients requiring invasive mechanical ventilation as compared to high-dose steroids.

Dexamethasone is on the list of essential medicines of the World Health Organization and is readily available worldwide at low cost.

## Figures and Tables

**Table 1:** A tabular presentation of the biochemical characteristics of the three groups of COVID-19 patients at the time of admission to the HDU categorized on the basis of their use of steroids. *p* value is used for expression of variables.

Variables	Dexa = 6 mg/Day (*N* = 176)	Dexa > 6 mg/Day (*N* = 48)	MP = 500 mg/Day (*N* = 209)	*p* Value
Age (years)	61.69 + 18.33	60.85 + 11.90	60.12 + 15.33	0.53
Comorbidities	33.5% (145/433)	7.4% (32/433)	37.4% (162/433)	0.07
PaO_2_/FiO_2_	163.28 + 86.88	193.64 + 95.82	179.62 + 89.51	0.17
Hemoglobin (g/dl)	11.43 + 2.12	11.16 + 2.17	11.22 + 2.39	0.98
WCC × 10^3^ cells/L	13.00 + 5.09	10.85 + 5.24	12.84 + 8.01	0.21
Lymphocytes (% age)	10.64 + 0.86	12.23 + 0.69	12.09 + 0.52	0.65
Platelets × 10^3^ cells/L	263.31 + 99.19	225.16 + 85.42	234.50 + 97.28	0.24
Urea (mmol/L)	11.60 + 9.52	12.75 + 3.17	11.01 + 7.87	0.31
Creatinine (umol/L)	267.60 + 35.45	234.57 + 22.98	288.02 + 59.77	0.34
D Dimers (ng/mL)	382.77 + 95.22	440.00 + 89.76	482.76 + 26.77	0.45
ALT (IU/L)	53.08 + 22.55	50.62 + 20.23	50.83 + 19.87	0.23
Albumin (g/L)	30.29 + 3.79	31.50 + 6.54	32.38 + 5.76	0.43
LDH (U/L)	492.56 + 158.06	503.33 + 185.32	444.59 + 194.76	0.08
Ferritin (ng/mL)	1829.78 + 168.64	932.21 + 103.52	1649.26 + 154.23	0.24
CRP (mg/L)	53.13 + 13.36	55.10 + 14.60	54.03 + 18.16	0.96
ProBNP (pg/mL)	5313.09 + 225.67	6067.98 + 348.97	6345.87 + 128.90	0.09
Trop T (ng/mL)	0.16 + 0.40	0.03 + 0.01	0.08 + 0.03	0.81
CPK (U/L)	213.57 + 28.94	234.55 + 23.96	233.00 + 24.05	0.46
CKMB (U/L)	36.42 + 21.89	34.54 + 20.65	27.97 + 13.36	0.03
PaO_2_	82.93 + 42.29	94.43 + 49.69	79.76 + 39.47	0.04

Creatinine kinase-MB [CKMB], lactate dehydrogenase [LDH], Pro-B-type natriuretic peptide [ProBNP], creatinine phosphokinase [CPK], white cell count [WCC], alanine aminotransferase [ALT], C-reactive protein [CRP], troponin T [Trop T], partial pressure of oxygen [PaO_2_], Dexamethasone [Dexa], Methylprednisolone [MP].

**Table 2:** A tabular representation of comparison of different modes of oxygen delivery at the time of admission to the HDU to 10 days treatment with three groups of steroids (Dexamethasone 6 mg/day, Dexamethasone > 6 mg/day, and Methylprednisolone 500 mg/day). *p* value is used for expression of variables.

Mode of Oxygen Delivery	At the Time of Admission	Dexa = 6 mg (*N* = 176)	Dexa > 6 mg (*N* = 48)	MP = 500 mg/Day (*N* = 209)	Contingency Co-Efficient	*p* Value
Oxygen < 10 L/min	1.8% (8/433)	22.9% (99/433)	1.4% (6/433)	21.9% (95/433)	48.19	<0.0001
Oxygen > 10 L/min	75.8% (328/433)	0	0.2% (1/433)	2.8% (12/433)		
NRM	12.2% (53/433)	5.5% (24/433)	0.9% (4/433)	4.8% (21/433)		
NIV	7.4% (32/433)	6% (26/433)	0.9% (4/433)	7.9% (34/433)		
Invasive Ventilation	2.8% (12/433)	6.2% (27/433)	7.6% (33/433)	10.9% (47/433)		

Non-rebreather mask [NRM], non-invasive ventilation [NIV], Dexamethasone [Dexa], and Methylprednisolone [MP].

**Table 3:** Variables predicting the survival of patients with COVID-19 infection according to Cox-proportional hazard regression analysis.

Variable	OR (95% CI)	*p* Value
Age	1.05 (1.03–1.22)	0.00
Co-morbid conditions	0.34 (0.14–0.84)	0.02
Number of days requiring oxygen	0.80 (0.73–0.88)	0.00
Number of days on ventilator support	0.91 (0.78–0.96)	0.00
Dexamethasone (6 mg/day)	0.11 (0.06–0.20)	0.00
Dexamethasone > 6 mg/day	1.08 (0.58–2.02)	0.80
Methylprednisolone (500 mg/day)	1.02 (1.00–1.31)	0.54

Odd ratio [OR]; confidence interval [CI].

## Data Availability

Not applicable.
